# Investigating the efficacy of skin swabbing as a minimally invasive method of genetic sampling from Indian amphibians

**DOI:** 10.1093/biomethods/bpag040

**Published:** 2026-07-11

**Authors:** G Vaibhavi, C K Aravind, Shashank K Borkar, Sona M Sebastian, Pavan Kumar Thunga, G Ravikanth, K V Gururaja, Priti Hebbar

**Affiliations:** Srishti Manipal Institute of Art Design and Technology, Manipal Academy of Higher Education, Manipal 576104, India; Manipal Institute of Technology Bengaluru, Manipal Academy of Higher Education, Manipal 576104, India; Srishti Manipal Institute of Art Design and Technology, Manipal Academy of Higher Education, Manipal 576104, India; Manipal Institute of Technology Bengaluru, Manipal Academy of Higher Education, Manipal 576104, India; Ashoka Trust for Research in Ecology and the Environment, Bengaluru 560064, India; Ashoka Trust for Research in Ecology and the Environment, Bengaluru 560064, India; Srishti Manipal Institute of Art Design and Technology, Manipal Academy of Higher Education, Manipal 576104, India; Manipal Institute of Technology Bengaluru, Manipal Academy of Higher Education, Manipal 576104, India

**Keywords:** anurans, tropical country, DNA amplification, DNA sequencing, Western Ghats

## Abstract

Amphibians are facing an unprecedented extinction crisis; 41% of the species are currently on the verge of extinction, making them among the most threatened vertebrates. One of the integral components in amphibian conservation is genetic research, which provides vital insights into their fitness and long-term persistence. Molecular studies on amphibians most often use invasive methods that are detrimental to them. Instead, minimally invasive methods such as cloacal, buccal, and skin swabbing can be effective. In this study, we collected skin swab samples from multiple anuran species in the Western Ghats biodiversity hotspot and the urban areas of Bengaluru, India, to assess the efficacy of skin swabs for generating genetic data without compromising animal welfare. Two extraction methods (salt vs Qiagen™ DNeasy kit) and two swab storage conditions (dry vs wet) were assessed. There was no significant difference in DNA yield between the two DNA extraction methods, whereas kit-based extractions consistently yielded DNA within the ideal purity range (A260/280 = 1.8–2.0). Swab storage did not significantly affect DNA concentration, but dry swabs provided significantly higher purity. Samples showed 86.36% amplification success on the gel, and 67.05% yielded high-quality DNA sequences suitable for downstream analyses. Our results demonstrate that minimally invasive skin swabs are a non-lethal technique that can be replicated in anurans using less DNA. This is the first study from the Indian tropical region to utilise multiple species of varying body sizes to demonstrate the feasibility of minimally invasive skin swabbing as a method for anuran research and conservation.

## Introduction

Amphibians are the most threatened vertebrates, with 41% of species at risk due to habitat loss, disease, and climate change [1]. Effective conservation of these taxa often depends on an integrated approach that involves molecular tools. These tools help to resolve cryptic diversity [2], assess population structure [3], and monitor genetic health [4]. Traditional genetic sampling techniques involve either lethal tissue extraction or invasive, non-lethal methods such as toe or tail clipping. Toe clipping consists of removing one toe or toe tip, while tail clipping requires excision of the amphibian’s tail or tail tip (of tadpoles in the case of anurans) [5]. While lethal methods provide high-quality DNA, they raise ethical concerns by inflicting injury, affecting the survival of amphibians [6–8]. Non-lethal but invasive methods like toe clipping may have neutral or negative effects on amphibians. A study by Narayan *et al.* [9] suggests toe clipping in amphibians induces a hormonal stress response, whereas a recent review by Zemanova *et al.* [10] suggests no effect. A study on another invasive method, such as tail clipping of tadpoles of anurans or salamanders, found no negative effects on their fitness and survival [11]. However, minimising impact on animals is a key refinement principle within the ‘3Rs’ (Replace, Reduce, Refine) framework, which aims to ensure that animal welfare during experimentation is optimised [12, 13]. Due to the growing emphasis on animal welfare, minimally invasive DNA sampling techniques have been advocated as more ethical than traditional lethal and invasive methods [14, 15].

Minimally invasive [16, 17] swabbing methods like buccal, cloacal, or skin swabbing although non-lethal, can have mixed benefits over traditional methods reported [14, 18–21]. Buccal and cloacal swabbing can be injurious to smaller organisms and may not be possible for all anurans, especially for the small-mouthed anurans [15, 22, 23]. On the contrary, skin swabbing is easy and less perilous to individuals [23]. Prunier *et al*. [15] used the skin swabbing method in two amphibian groups, confirming its utility for non-lethal genetic studies. Skin swabbing has also been used in conservation genetics and phylogeographical studies in amphibians [24, 25]. However, they yield lower DNA quantity and purity compared to buccal swabs. This could be dependent on amphibians’ size and skin texture [23]. For example, in large alpine newt skin swabbing was an excellent alternative to buccal swabbing, providing good genotyping success [15]. Yet, a study on small-sized dendrobatid frogs yielded low DNA and less genotyping success [23]. Not just skin texture, type of extraction method and storage medium also influence DNA yield. While there are no such studies using skin swabs, a study on the efficacy of buccal swabs for DNA recovery in anurans suggested ethanol as a better storage medium than a non-alcoholic lysis buffer, and Qiagen™ kit DNA extraction showed higher purity than the salt extraction method [17]. Such results can be attributed to the presence of skin alkaloids and skin microbiota in amphibians [17, 23], which hinder the successful application of skin swabbing in amphibian genetic studies. Among the minimally invasive methods, skin swabbing is less harmful to amphibians [26]. Therefore, there is a need to understand the efficacy of skin swabbing in anurans by comparing extraction methods, storage media, and testing the utility in various anuran genera.

The efficacy of skin swabbing for amphibians remains underexplored in tropical landscapes with heterogeneous climatic conditions. Tropical landscapes have high amphibian diversity [27]; hence, it is essential to adopt minimally invasive methods that can support amphibian welfare without hindering the research. In this study, we tested the efficacy of skin swabbing as a non-lethal, minimally invasive method for DNA recovery from Indian anurans. We assess DNA yield, purity, and amplification success from skin swabs, using different extraction methods (kit and salt) and storage methods. We investigate the influence of storage medium on DNA yield and assess the overall success rate of amplification and downstream molecular sequencing. We also test the influence of body size on DNA yield. To our knowledge, this is the first study to investigate the efficacy of skin swabs for genetic analyses of anurans from India and the second study from intertropical climatic conditions [17]. By establishing the reliability of skin swabbing in the intertropical climate conditions where optimising such protocols can be difficult due to heterospecific conditions, this work aims to promote a non-lethal and minimally invasive sampling technique that will help in taxonomy, genetic surveys, conservation, and evolutionary research without compromising amphibian welfare.

## Materials and methods

### Study area and sampling

**Figure 1:**
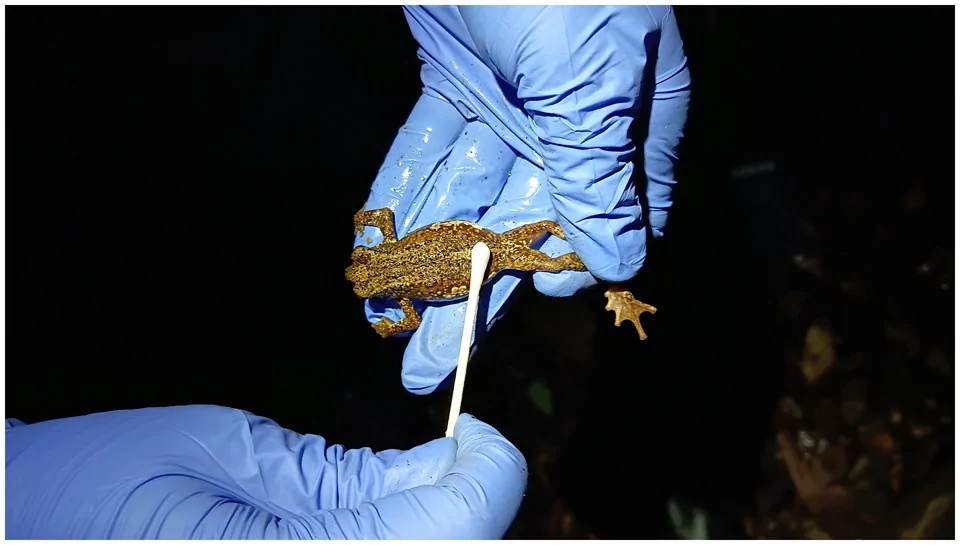
Skin swabbing of *Pedostibes tuberculosus* in the field

Surveys and sampling were conducted between May 2024 and February 2026 across the Western Ghats biodiversity hotspot and the urban regions of Bengaluru, Karnataka. The work was undertaken as part of ongoing research studies on Indian anurans. A total of 88 skin swab samples were collected opportunistically from anurans belonging to seven genera: *Duttaphrynus*, *Euphlyctis*, *Indirana*, *Microhyla*, *Minervarya*, *Nyctibatrachus*, and *Pedostibes* (Table 1). The details of the location are provided in Supplementary 1. Skin swabbing was performed using commercially available sterile, round, cotton-tipped Hi Media® swabs (130 mm length, tip width = 3 mm, tip length = 15 mm). Each swab was gently rolled over the individual’s skin from snout to vent along the dorsal and ventral surfaces, armpit to the tip of each fore-limb finger, and thigh to the tip of each hind-limb toe (Fig. 1). Each full, continuous roll with gentle pressure was counted as one ‘stroke.’ A total of 60 strokes were applied per individual based on preliminary analysis. However, in two cases, the individuals jumped off the hand, hence we could swab 34 and 25 strokes, respectively. To maintain skin moisture during swabbing, distilled or sterile water was applied to the skin surface. After swabbing, individuals were released at the point of capture and monitored for 15–20 minutes to assess any visible behavioural or stress responses. No adverse effects were observed in any individuals post-release. Swabs were preserved using two storage methods: (i) wet swabs stored in 90% ethanol, and (ii) dry swabs stored in sterile, dry Eppendorf microcentrifuge tubes. All samples were kept at −20°C until further processing and DNA extraction.

**Table 1:** Details of the skin swab samples collected.

Serial number	Genus	Location	State	Total number of samples collected
1	*Euphlyctis*	Western ghats	Karnataka	2
2	*Duttaphrynus*	Bengaluru	Karnataka	10
3	*Indirana*	Western ghats	Karnataka and Maharashtra	20
				
4	*Microhyla*	Bengaluru	Karnataka	4
5	*Minervarya*	Bengaluru	Karnataka	2
6	*Nyctibatrachus*	Western ghats	Karnataka and Kerala	28
				
7	*Pedostibes*	Western ghats	Karnataka	22

### DNA extraction, amplification, and sequencing

Two DNA extraction methods were tested for this study: (i) a commercial kit-based approach using the DNeasy Blood & Tissue kit (Qiagen™ Germany) following the manufacturer’s protocol, and (ii) a salt extraction protocol following the method by Vences *et al.* [28]. As part of both protocols, samples were left to digest overnight for approximately 16 hours.

### Amplification and sequencing

A partial region of 16S rRNA: 16Sar-L (5′-CGCCTGTTTATCAAAAACAT-3′) and 16Sbr-H (5′-CCGGTCTGAACTCAGATCACGT-3′) [29] were used. Polymerase chain reactions (PCR) were prepared in a total volume of 25 µl, typically containing 1–1.5 µl of extracted DNA, 3–10 µl of nuclease-free water, 10–11 µl of Ampliqon master mix (Cat. No: A180303), and 1 µl each of forward and reverse primers. Thermal cycling conditions were as follows: initial denaturation at 95°C for 5 minutes; 30–40 cycles of denaturation at 95°C for 30–45 seconds, annealing at 55°C for 1–1.5 minutes, and extension at 72°C for 1.5–2 minutes; followed by a final extension at 72°C for 5–7 minutes and a hold at 10°C. For samples that failed to amplify at 55°C, alternative annealing temperatures of 45°C, 52°C, and 53°C were used. The number of cycles was also adjusted between 30 and 40 to optimise band intensity. A few samples showed non-specific bands. For such samples, a touchdown PCR (TD-PCR) protocol was used [30, 31]. TD-PCR was run at three phases: Phase 1 with one cycle of initial denaturation at 95°C for 5 min, seven cycles of denaturation at 95°C for 30 seconds, annealing at 62°C for 40 seconds and elongation at 72°C for 40 seconds; Phase 2 with one cycle of denaturation at 95°C for 40 seconds, 26–29 cycles annealing at 55°C for 55 seconds and elongation at 72°C for 55 seconds, Phase 3 with final elongation at 72°C for 5 minutes and hold at 10°C was used.

DNA yield and purity were assessed using a NanoDrop Lite Plus micro–UV Spectrophotometer. Measurements of DNA concentration (ng/µl) and purity ratios (A260/280 and A260/230) were recorded in triplicate for each sample. For kit-extracted samples, the NanoDrop was blanked using the elution buffer (AE buffer) provided in the kit. For samples extracted using the salt extraction method, nuclease-free water was used as a blank. Amplification success was assessed on a 2% agarose gel electrophoresis. Successfully amplified PCR products were sent for sequencing at Barcode Biosciences, Bengaluru. Species identity was confirmed through the Nucleotide Basic Local Alignment Search Tool (BLAST) searches against the National Center for Biotechnology Information nucleotide database. The sequences were deposited in GenBank under accession numbers PX43971-PX439734 and PZ322345-PZ322379.

### Statistical analysis

We conducted normality tests for the data using the Shapiro–Wilk test. We used a *t*-test for log-transformed normally distributed data [for DNA concentration (ng/µl): salt and kit; dry and wet; Table 2] with equal sample sizes to compare the two independent groups. We used the Mann–Whitney U test when the assumptions of normality were not met [even after log transformation for DNA purity (260/280): salt, kit, dry and wet; Table 3]. All statistical analyses were conducted, and graphs were plotted using the *stats* and *ggplot* packages in R/RStudio© [32].

**Table 2:** Results of Shapiro–Wilk’s test for log-transformed DNA concentration(ng/µl) values.

Shapiro–Wilk’s test	*N*	W	*P*-value
Salt extraction	44	0.99	.86
Kit extraction	44	0.96	.11
Dry storage	44	0.97	.31
Wet storage	44	0.98	.50

**Table 3:** Results of the Shapiro–Wilk test conducted for DNA purity(A260/280).

Shapiro–Wilk’s test	*N*	W	*P*-value
Salt extraction	44	0.95	.05
Kit extraction	44	0.98	.77
Dry storage	44	0.98	.49
Wet storage	44	0.95	.06

## Results

**Figure 2:**
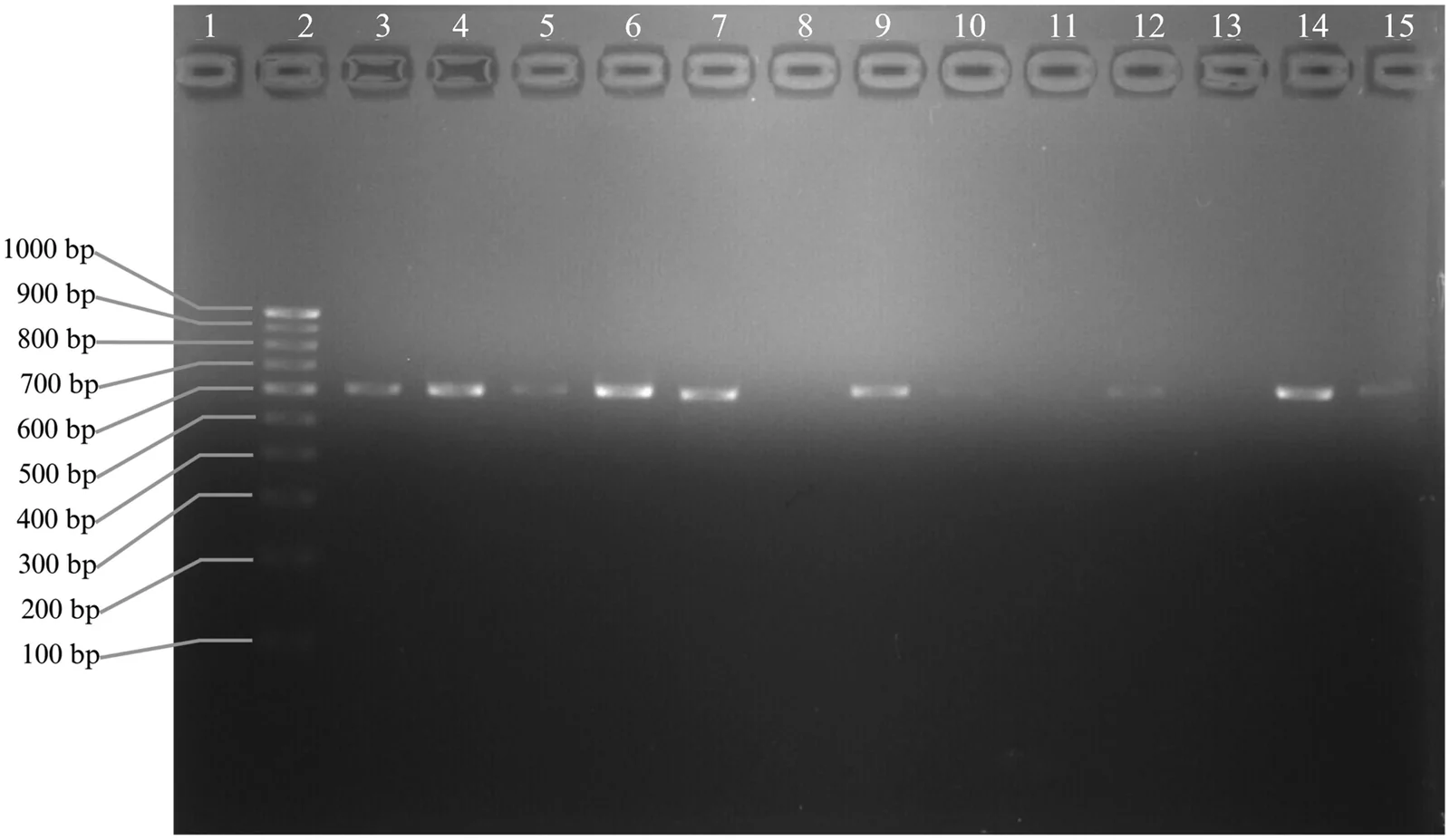
PCR amplification of skin swab from *Pedostibes tuberculosus* (PE). lane 2: 100 bp ladder. Lanes 3–15: amplified samples (PE3, PE4, PE6, PE7, PE2, PE5, PE8, PE10, PE9, PE1, PE11). Lanes 1, 7, and 13 are not a part of this study

**Figure 3:**
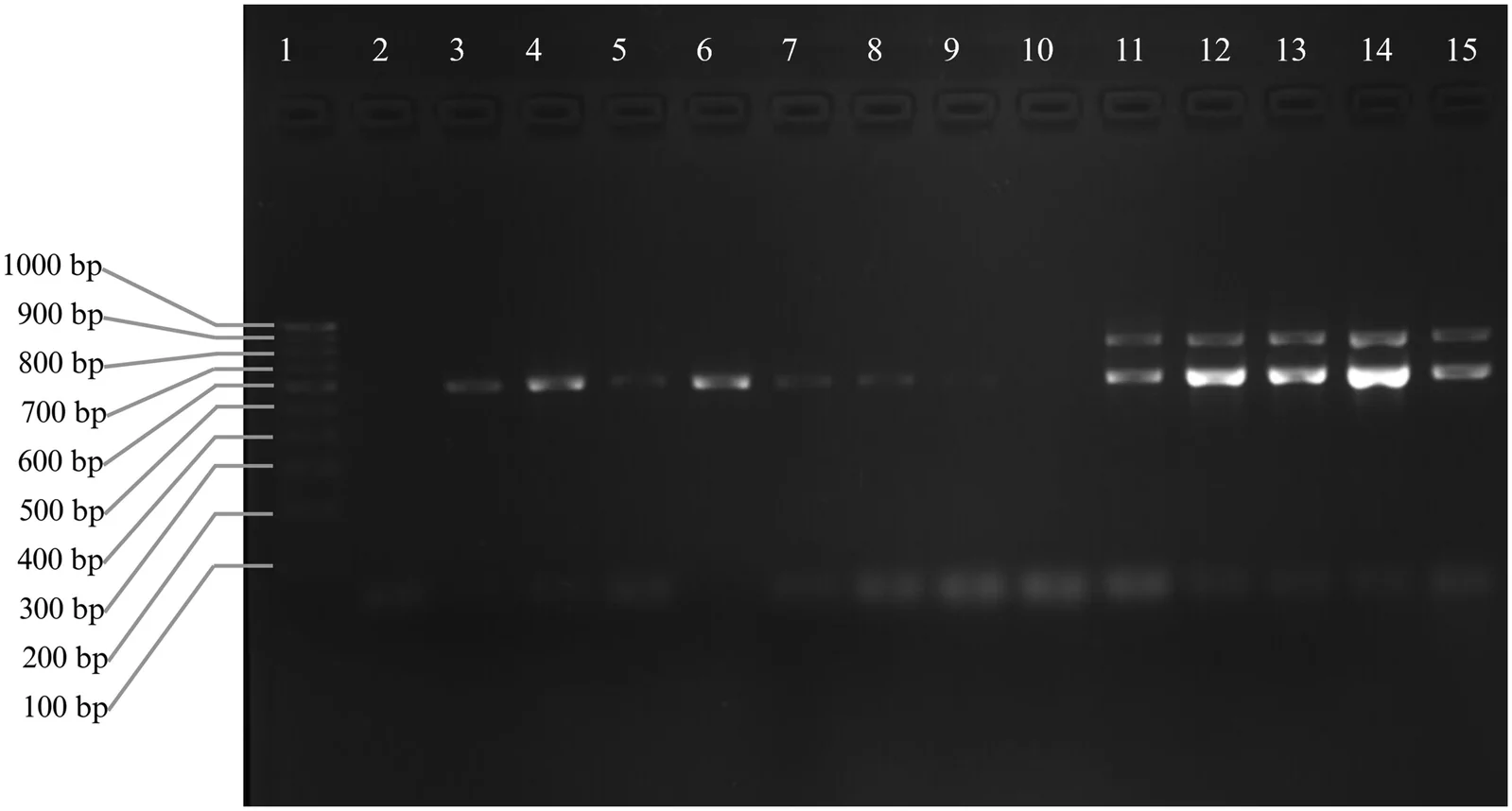
PCR amplification of skin swab from genus *Indirana* (IN). Lane 1: DNA ladder. Lanes 2–10: amplified samples (IN15, IN16, IN2, IN3, IN4, IN5, IN08, IN01, IN09). Lanes 11–15 were not a part of this study

**Figure 4:**
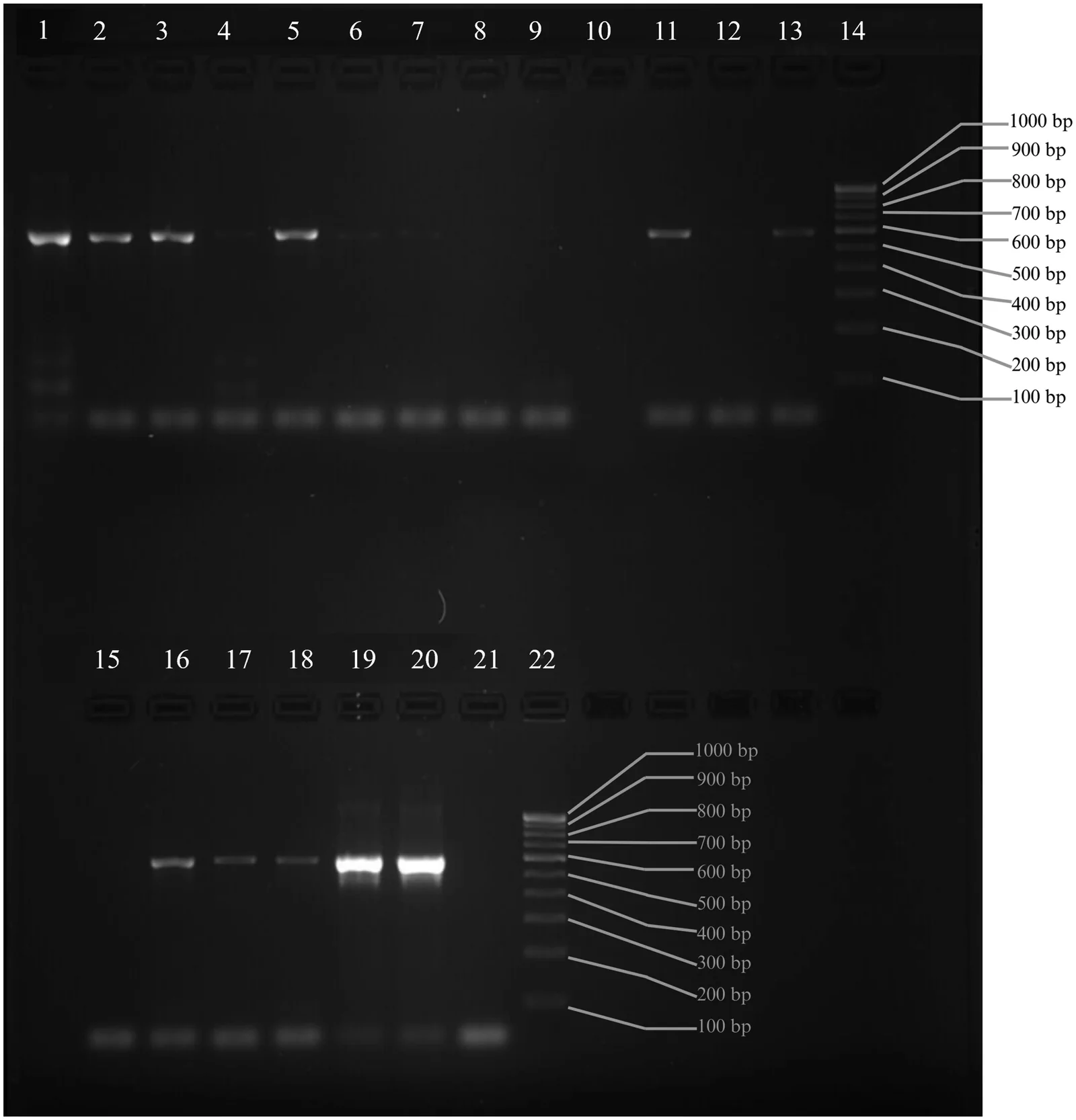
PCR amplification of skin swab from *Minervarya* (MN), *Euphlyctis* (EU), and *Nyctibatrachus* (NY). Lane 3: NY2; lane 5: MN2; lane 11: EU2; lane 14: ladder; lane 16: NY8; lane 17: NY5; lane 18: NY6; lane 21: negative control; lane 22: ladder. Lanes 1, 2, 4, 6–10, 12–13, 15, 19–20 were not part of this study

**Figure 5:**
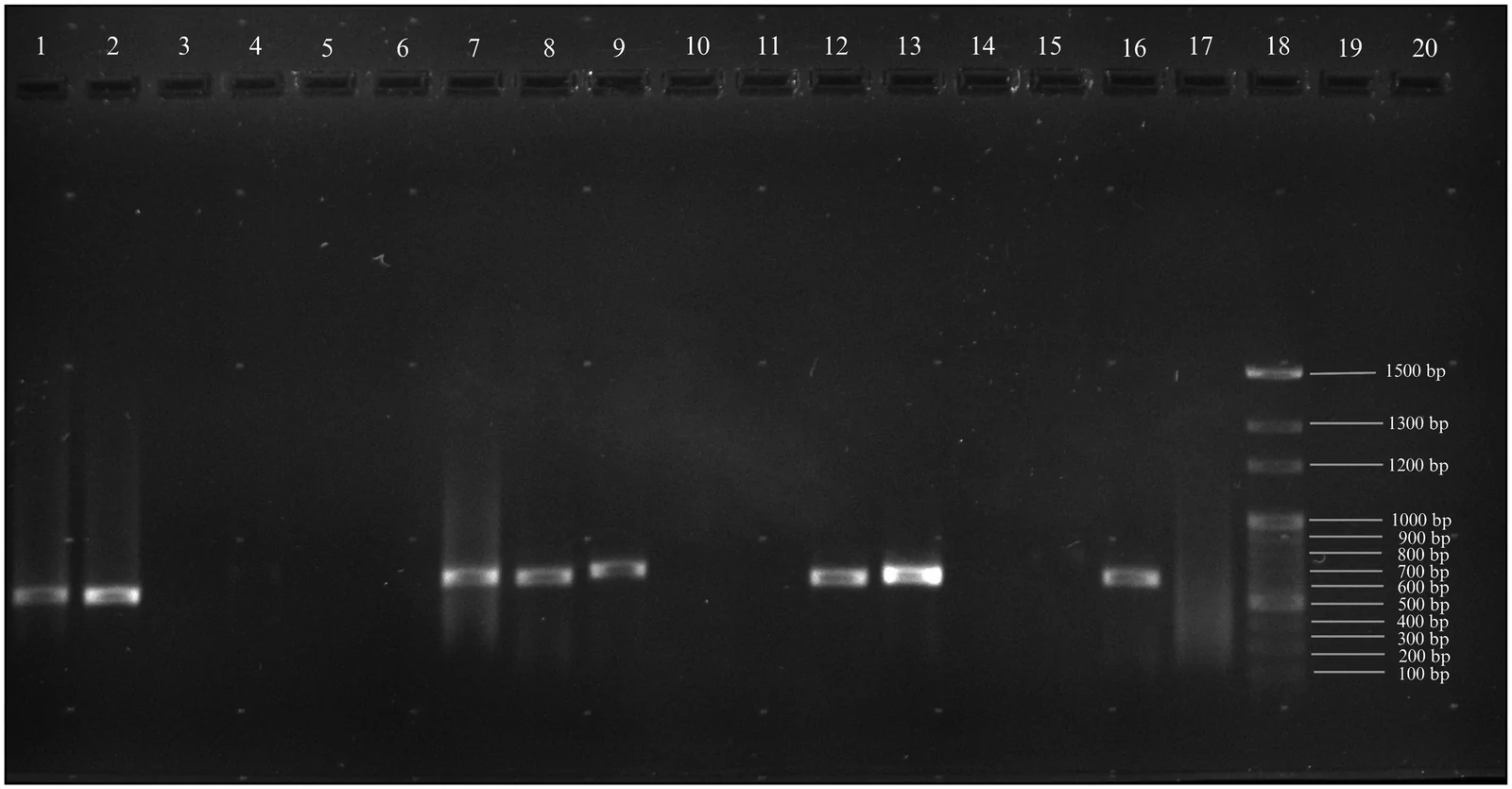
PCR amplification of skin swab from genus *Nyctibatrachus* (NY), *Indirana* (IN), *Pedostibes* (PE), and *Duttaphrynus* (DU). Lanes 1, 2: NY16, NY26; lane 3: IN 20; lane 4—PE21; lanes 5–11: DU4, DU5, DU6, DU7, DU8; DU9, DU10; lanes 12–13: NY11, NY18; lanes 14–15 were not part of the study. Lane 16–17: positive and negative sample respectively; lane 18 is ladder

**Figure 6:**
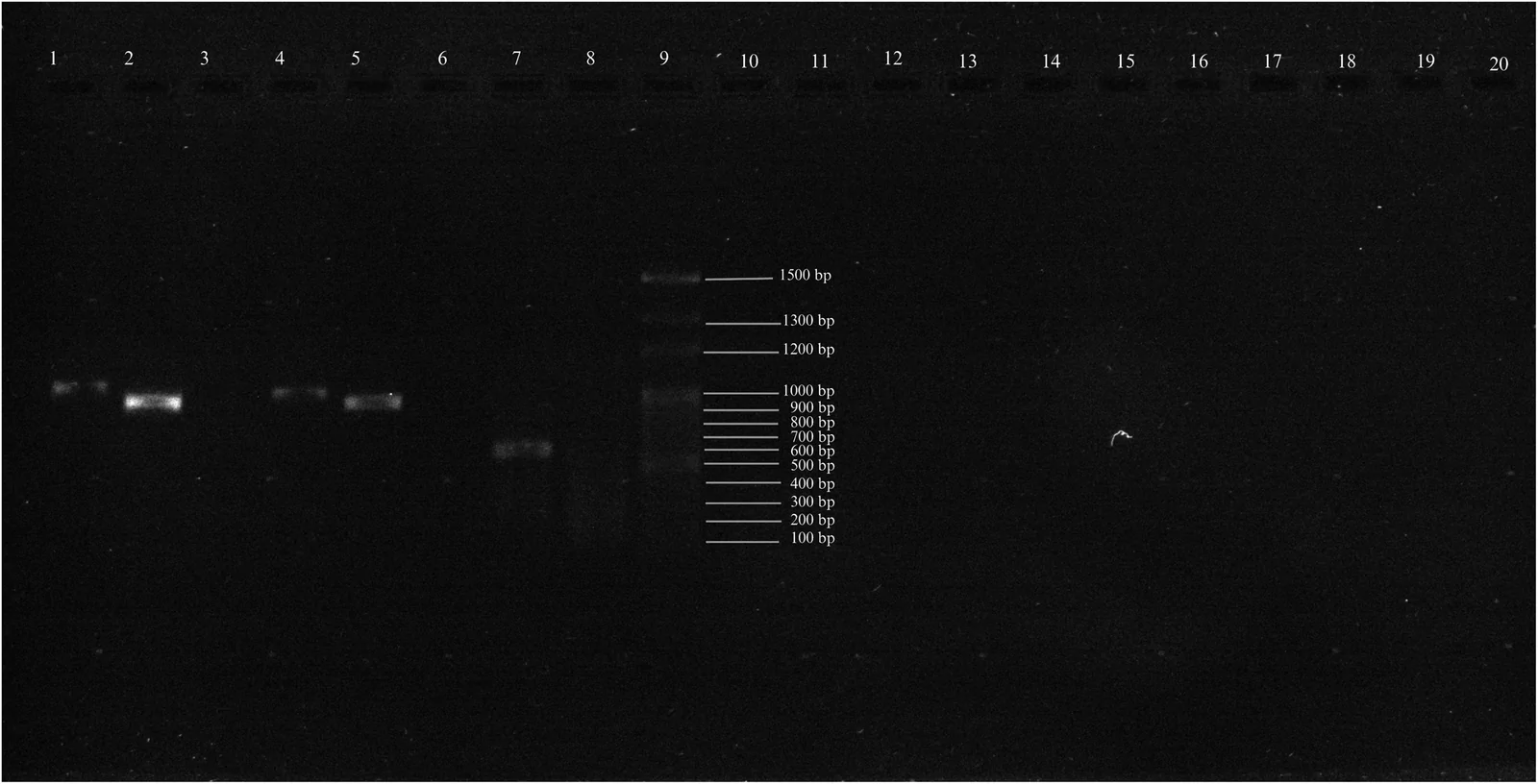
PCR amplification of skin swab from genus *Microhyla* (MC). Lane 5: MC4, lanes 1–4, 6–8 were not part of the study, lane 9: Ladder, lanes 10–20 were not used.

We swabbed 88 individuals of anurans belonging to seven genera. Although protocol optimisation was required to achieve amplification across samples, we successfully amplified DNA from various species, as shown in the gel electrophoresis images (Figs 2–6). DNA from *Pedostibes tuberculosus* (Fig. 2), *Indirana* species (Fig. 3) and *Minervarya, Euphlyctis*, *Nyctibatrachus* (Fig. 4), *Duttaphrynus* (Fig. 5), and *Microhyla* species (Fig. 6) showed clear amplification bands.

### A comparative analysis of DNA yield and purity from different extraction and storage methods

#### DNA yield

**Figure 7:**
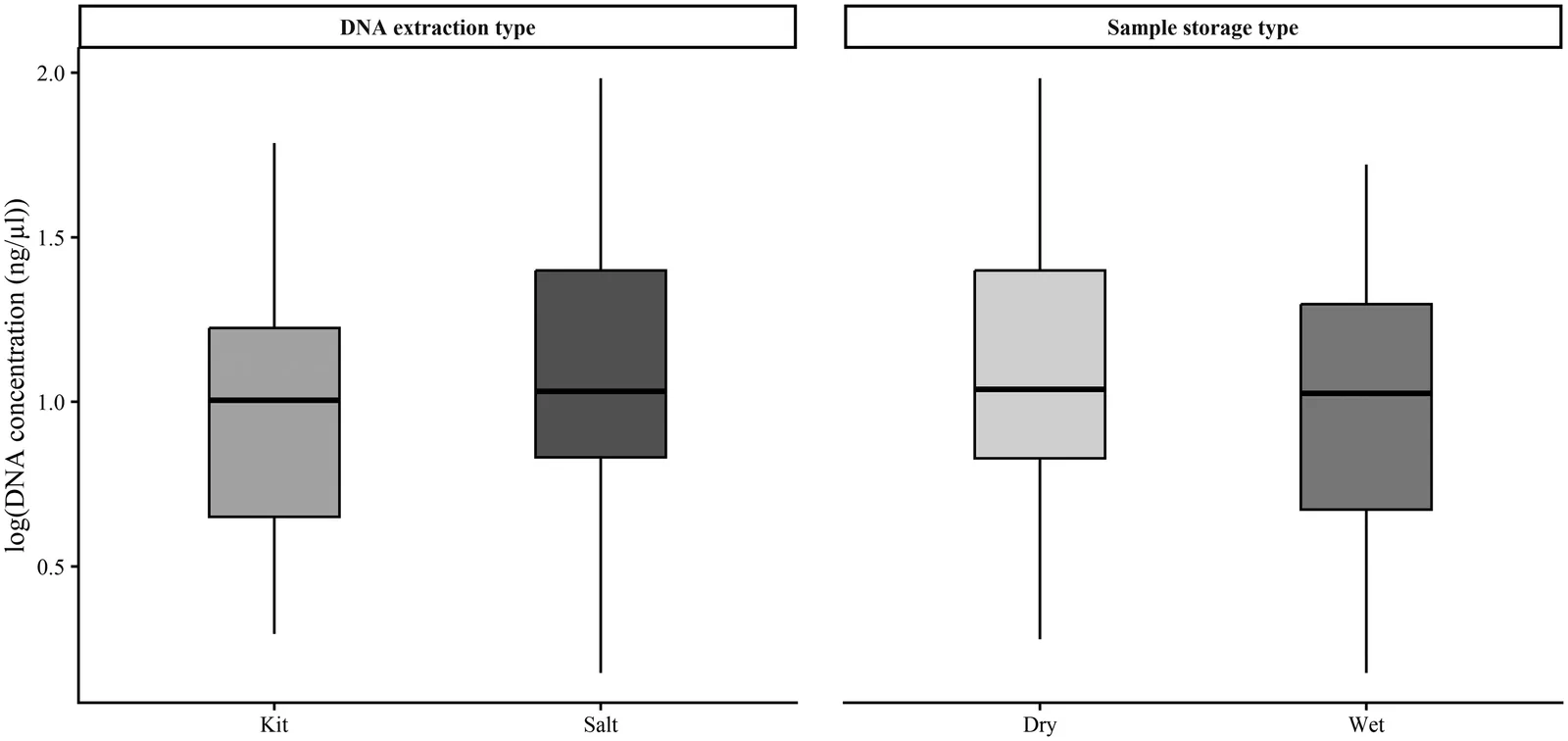
Box plot comparing the DNA concentration of two extraction methods (kit- and salt-extraction method) and the two storage methods (dry and wet storage).

DNA concentration obtained from the salt extraction method ranged from 1.5 to 96.2 ng/µl, while the commercial kit extraction ranged from 2.53 to 29.60 ng/µl. The t-test showed no significant difference between the DNA concentrations obtained using the salt and kit extraction methods (*t* = −0.66, *P* > 0.05). Mean yields were comparable between the salt [Mean ± standard deviation (SD) = 1.07 ± 0.44, *N* = 44] and kit extraction methods (Mean ± SD) = 1.01 ± 0.40, *N* = 44; Fig. 7, Table 4).

**Table 4:** Results of the parametric test conducted for log-transformed DNA concentration(ng/µl) values.

	Statistical test	*N*	*t*	*P*-value	**Mean** **±** **SD**
Salt vs. kit	*t*-test, variance equal	Salt = 44, Kit = 44	−0.66	.51	Kit = 1.01 ± 0.40, salt = 1.07 ± 0.44
Dry vs. wet	*t*-test, variance equal	Dry = 44, Wet = 44	0.75	.45	Dry = 1.07 ± 0.45, wet = 1.00 ± 0.39
Salt-wet vs. salt-dry	*t*-test, variance equal	Salt-Wet = 22, Salt-Dry = 22	−0.60	.55	Salt-wet = 1.11 ± 0.39, salt-dry = 1.03 ± 0.50
Kit-wet vs kit-dry	*t*-test, variance equal	Kit-Wet = 22, Kit-Dry = 22	1.83	.07	Kit-wet = 0.90 ± 0.38, kit-dry = 1.12 ± 0.40

DNA concentration from the dry storage method ranged from 1.9 to 96.2 ng/µl, while the DNA concentration from the wet storage method ranged from 1.5 to 52.63 ng/µl. Mean yields were comparable between dry storage (Mean ± SD = 1.07 ± 0.45, *N* = 44) and wet storage (Mean ± SD = 1.01 ± 0.39, *N* = 44), with no significant difference (*t* = 0.75, *P* > 0.05; Fig. 7, Table 4).

#### DNA purity

**Figure 8:**
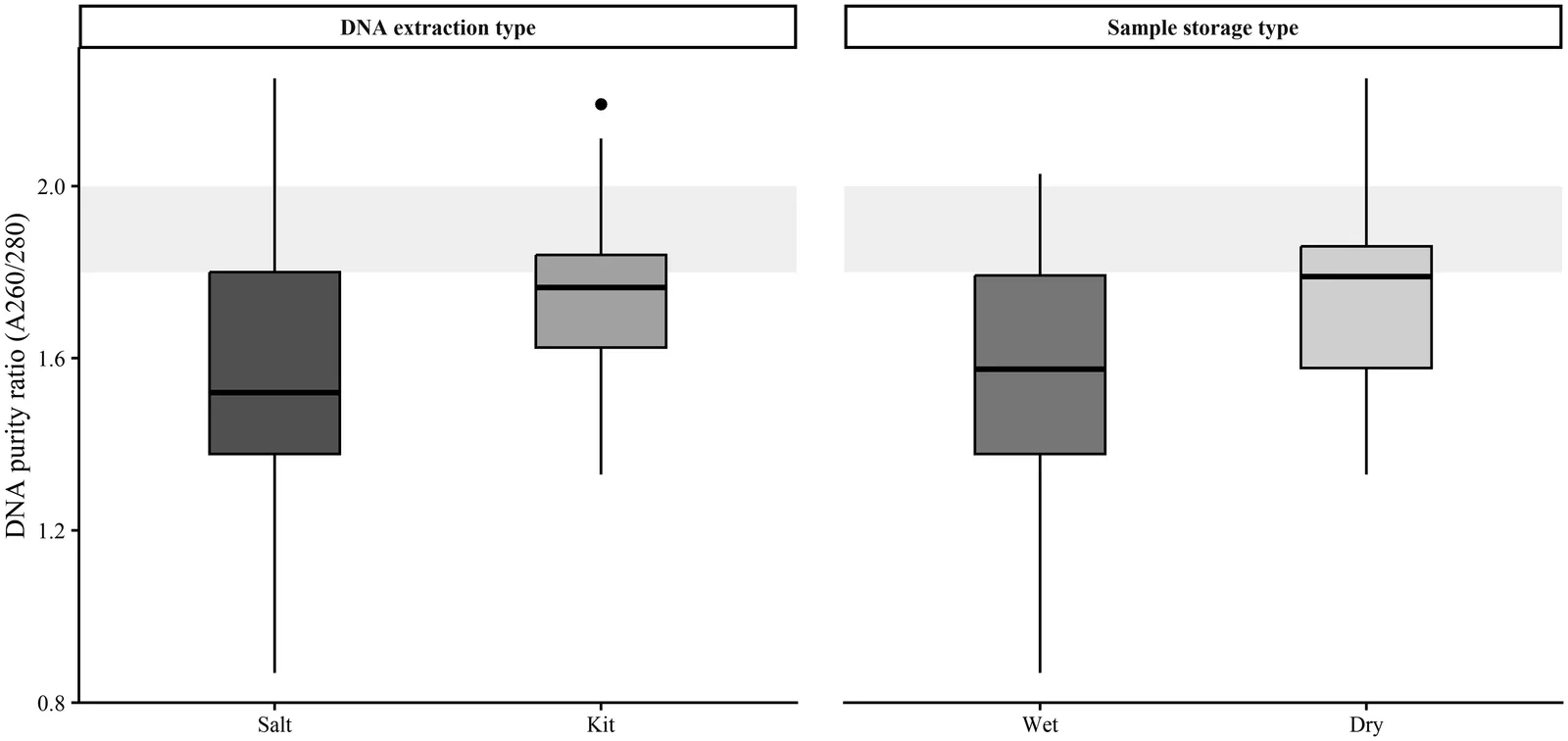
Box plot comparing the DNA purity ratio (A260/280) of two extraction methods (kit- and salt-extraction method) and the two storage methods (dry and wet storage).

A significant difference was observed between salt-extracted and kit-extracted DNA purity values (Mann–Whitney *U* test, W = 1318.5, *P* < 0.05; Fig. 8, Table 5). Salt-extracted DNA had lower purity values (Mean ± SD = 1.59 ± 0.28, Median = 1.52, *N* = 44) compared to kit-extracted DNA (Mean ± SD = 1.74 ± 0.19, Median = 1.77, *N* = 44), with the latter falling closer to the ideal range of 1.8–2.0.

**Table 5:** Results of the non-parametric test conducted for DNA purity(A260/280).

DNA purity ratio (260/280)	Statistical test	*N*	W	*P*-value	**Mean** **±** **SD**
Salt vs. kit	Mann–Whitney *U* test	Salt Mann–Whitney *U* test = 44, Kit = 44	1318.5	.003	Salt = 1.588 ± 0.28, kit = 1.74 ± 0.19
Dry vs. wet	Mann–Whitney *U* test	Dry = 44, Wet = 44	1346.5	.002	Dry = 1.75 ± 0.23, wet = 1.58 ± 0.24
Salt-wet vs. salt-dry	Mann–Whitney *U* test	Salt-wet = 22, Salt-dry = 22	399.5	.0002	Salt-wet = 1.44 ± 0.22, salt-dry = 1.74 ± 0.26
Kit-wet vs kit-dry	Mann–Whitney *U* test	Kit-wet = 22, Kit-dry = 22	276	.4314	Kit-wet = 1.71 ± 0.18, kit-dry = 1.76 ± 0.20

Dry and wet storage methods showed a significant difference with reference to DNA purity ratio (W = 1346.5, *P* < 0.05, Table 5). DNA from dry swabs exhibited higher purity (Mean ± SD = 1.75 ± 0.23, Median = 1.79, *N* = 44) than wet swabs (Mean ± SD = 1.58 ± 0.24, Median = 1.58, *N* = 44), with values from dry swabs aligning more closely with the optimal purity range (Fig. 8).

### A comparative analysis of DNA yield and purity from salt-extracted dry vs wet storage, kit-extracted dry vs wet storage methods

#### DNA yield

**Figure 9:**
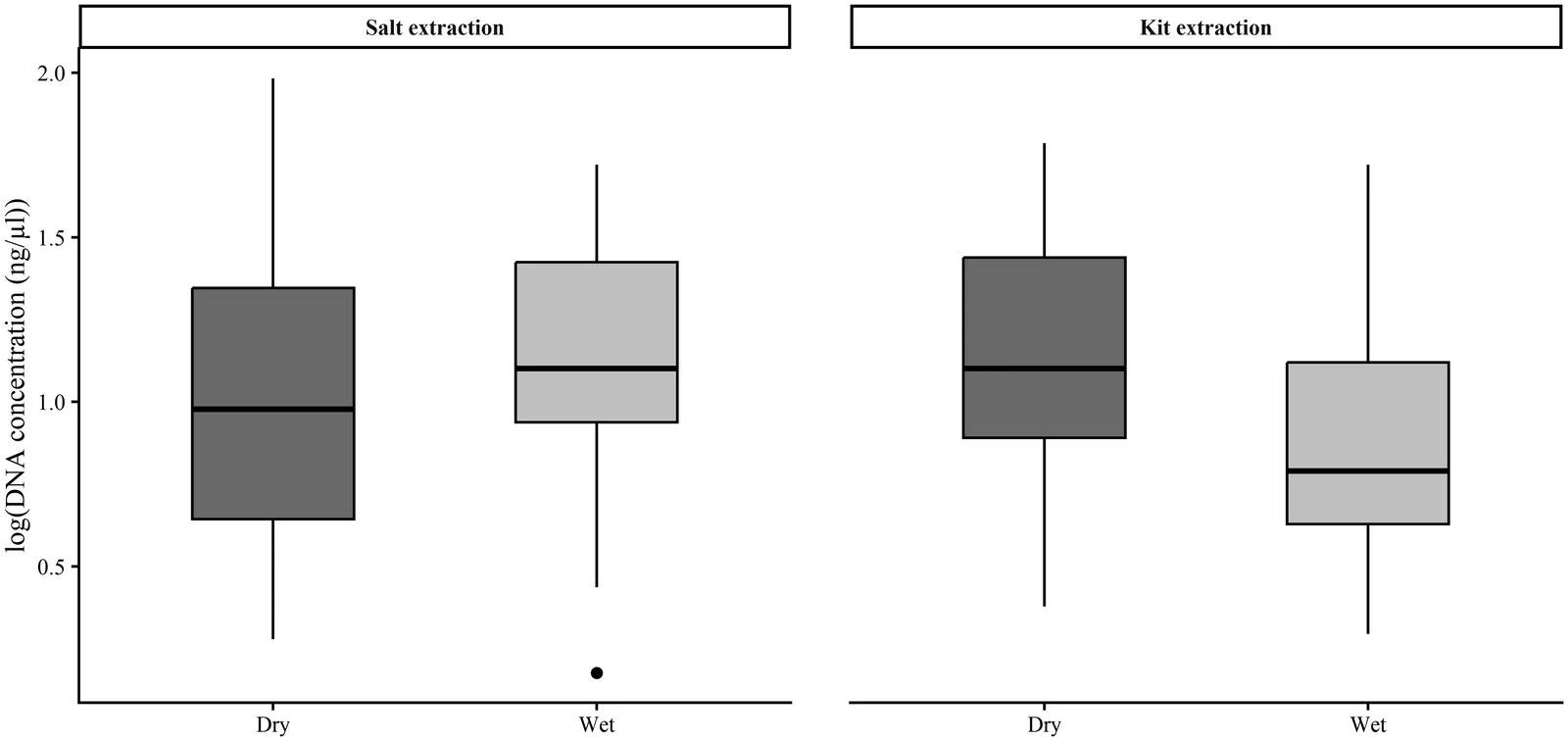
Box plot comparing the DNA concentration of salt-extracted dry vs. wet storage and kit-extracted dry vs. wet storage.

We compared salt-extracted dry storage (Mean ± SD = 1.03 ± 0.50, *n* = 22) vs wet storage (Mean ± SD = 1.11 ± 0.39, *n* = 22) samples for the DNA concentration (ng/µl) obtained. t-test showed no significant difference (*t* = −0.60, *P* > 0.05). In the case of kit extraction, the mean of dry storage samples (Mean ± SD = 1.12 ± 0.40, *n* = 22) was slightly higher than that of wet storage samples (Mean ± SD = 0.90 ± 0.79, *n* = 22, Fig. 9, Table 3). However, there was no statistically significant difference between them (*t* = 1.83, *P* > 0.05).

#### DNA purity

**Figure 10:**
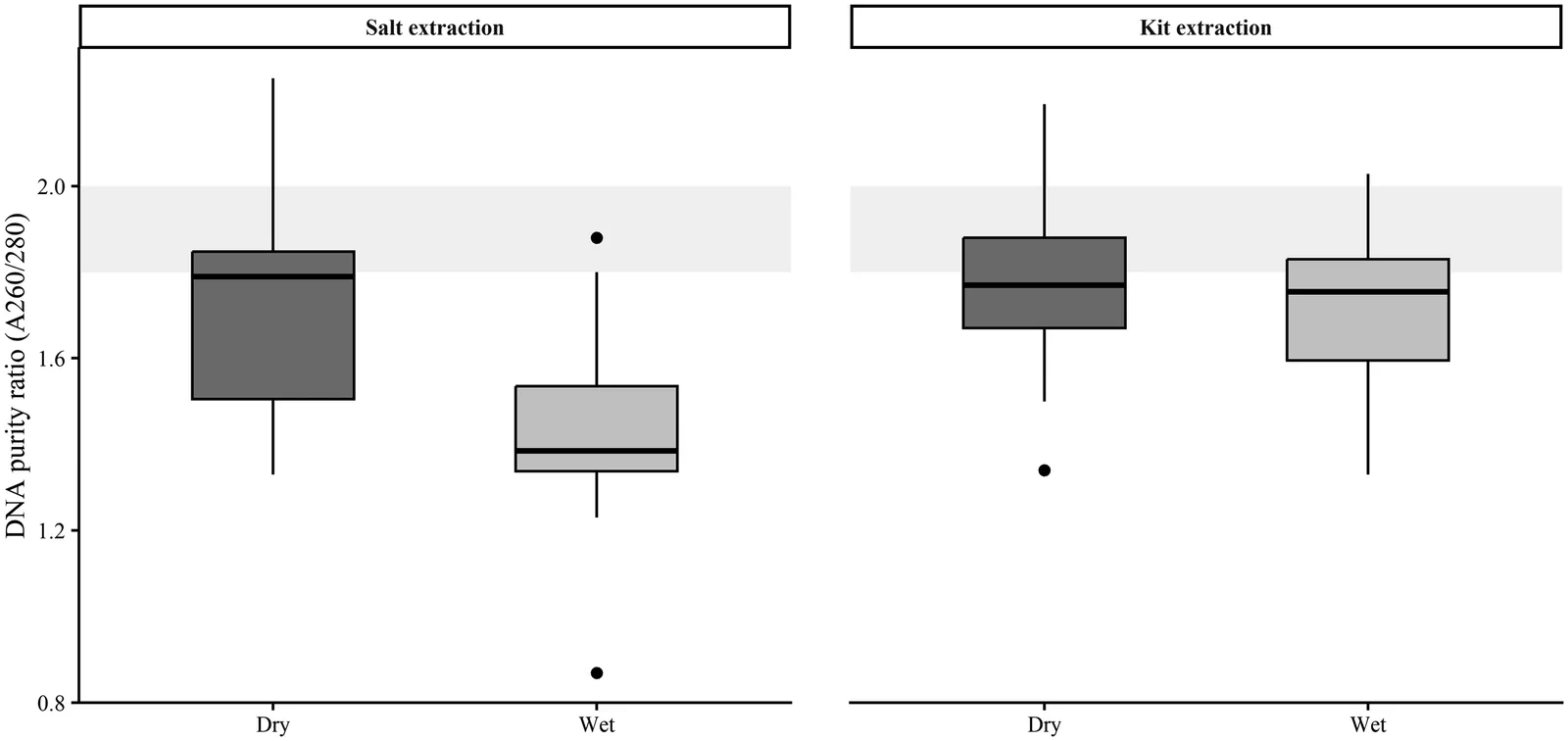
Box plot comparing the DNA purity ratio (A260/280) of salt-extracted dry vs wet storage and kit-extracted dry vs wet storage.

We compared the purity ratio between salt-extracted, dry vs wet storage, and kit-extracted, dry vs wet storage samples (Fig. 10). The results showed statistically significant difference (W = 399.5, *P* < 0.05) between dry storage (Mean ± SD = 1.74 ± 0.26, Median = 1.79, *n* = 22) and wet storage samples (Mean ± SD = 1.44 ± 0.22, Median = 1.39, *n* = 22). In case of kit extraction, dry storage (Mean ± SD = 1.77 ± 0.20, Median = 1.77, *n* = 22) and wet storage (Mean ± SD = 1.72 ± 0.18, Median = 1.76, *n* = 22) samples were not significantly different (W = 276, *P* > 0.05, Fig. 10, Table 4).

### PCR amplification and 16S rRNA sequencing success

**Figure 11:**
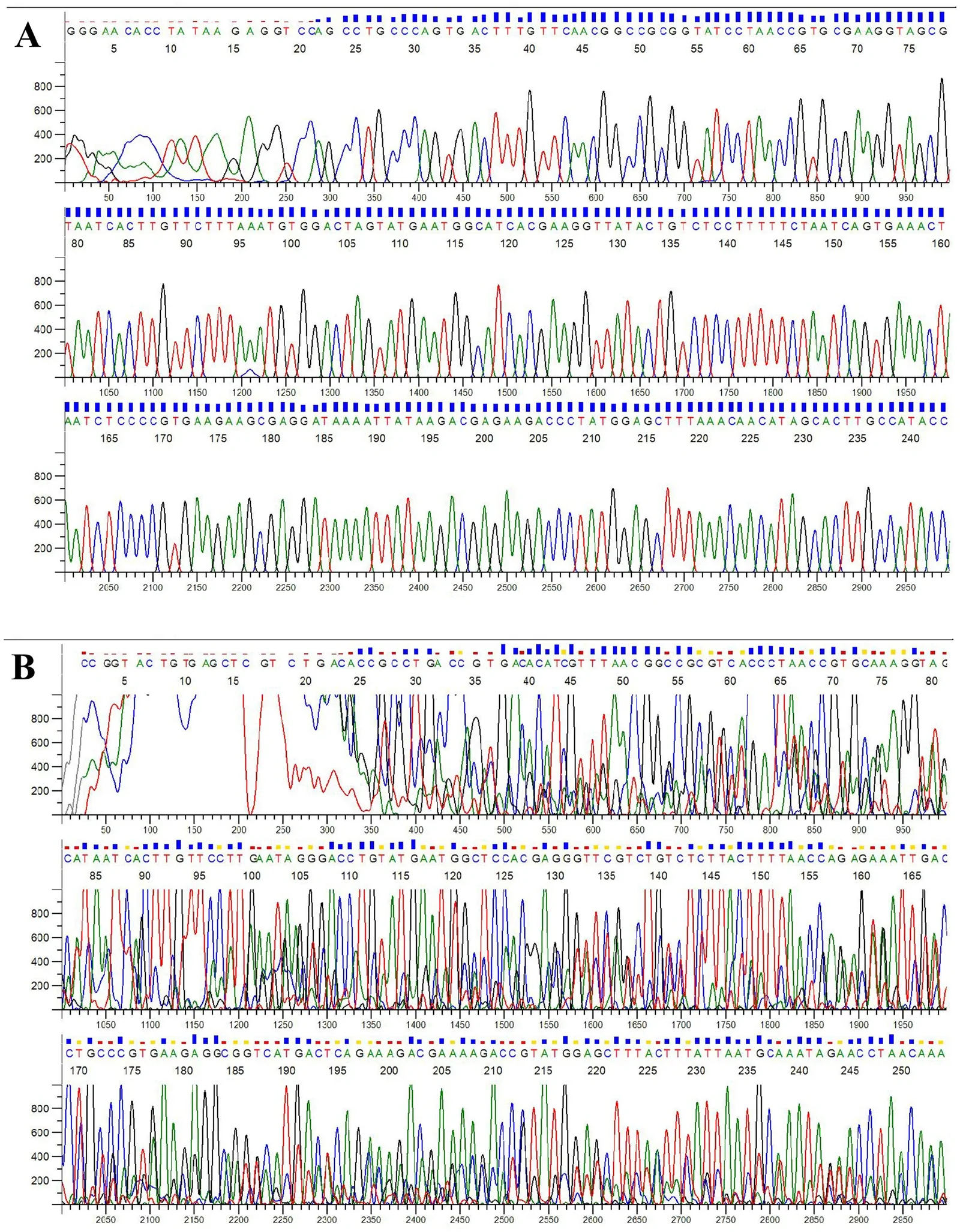
Representatives of (A) clean, readable 16S chromatogram (sample: PE3), (B) messy 16S chromatogram (sample: IN15).

A total of 88 skin swab samples were analysed, of which 76 (N = 88, 86.36%) showed amplification on the gel, and 59 (N = 88, 67.05%) yielded a clean, readable 16S chromatogram after sequencing. A clean, readable chromatogram is shown alongside a messy sequence from DNA extracted from skin swabs in Fig. 11.

**Figure 12:**
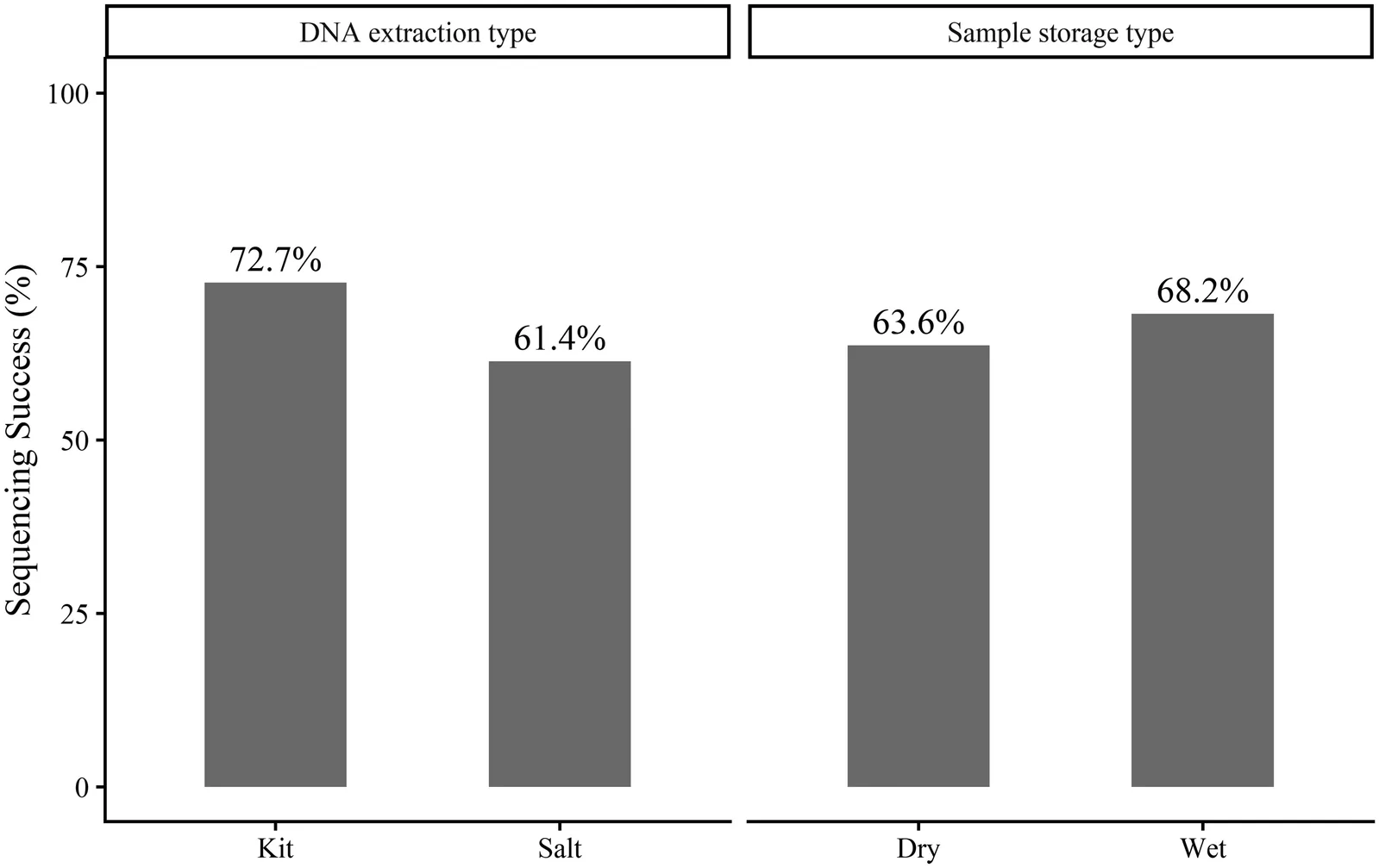
Sequencing success (%) of DNA samples under different DNA extraction and storage methods.

Under the salt extraction method, 39 out of 44 samples were successfully amplified on the gel (88.64%), and 27 out of 44 samples yielded clean sequencing reads (61.36%). Similarly, under the kit extraction method, 37 of 44 samples were successfully amplified on the gel (84.09%), and 32 of 44 samples yielded clean sequencing reads (72.73%). Under the dry storage method, 39 out of 44 samples successfully amplified on the gel (88.64%), and 29 out of 44 samples yielded clean sequencing reads (65.91%). Similarly, under the wet storage method, 37 out of 44 samples successfully amplified on the gel (84.09%), and 30 out of 44 samples yielded clean sequencing reads (68.18%; Fig. 12).

**Figure 13:**
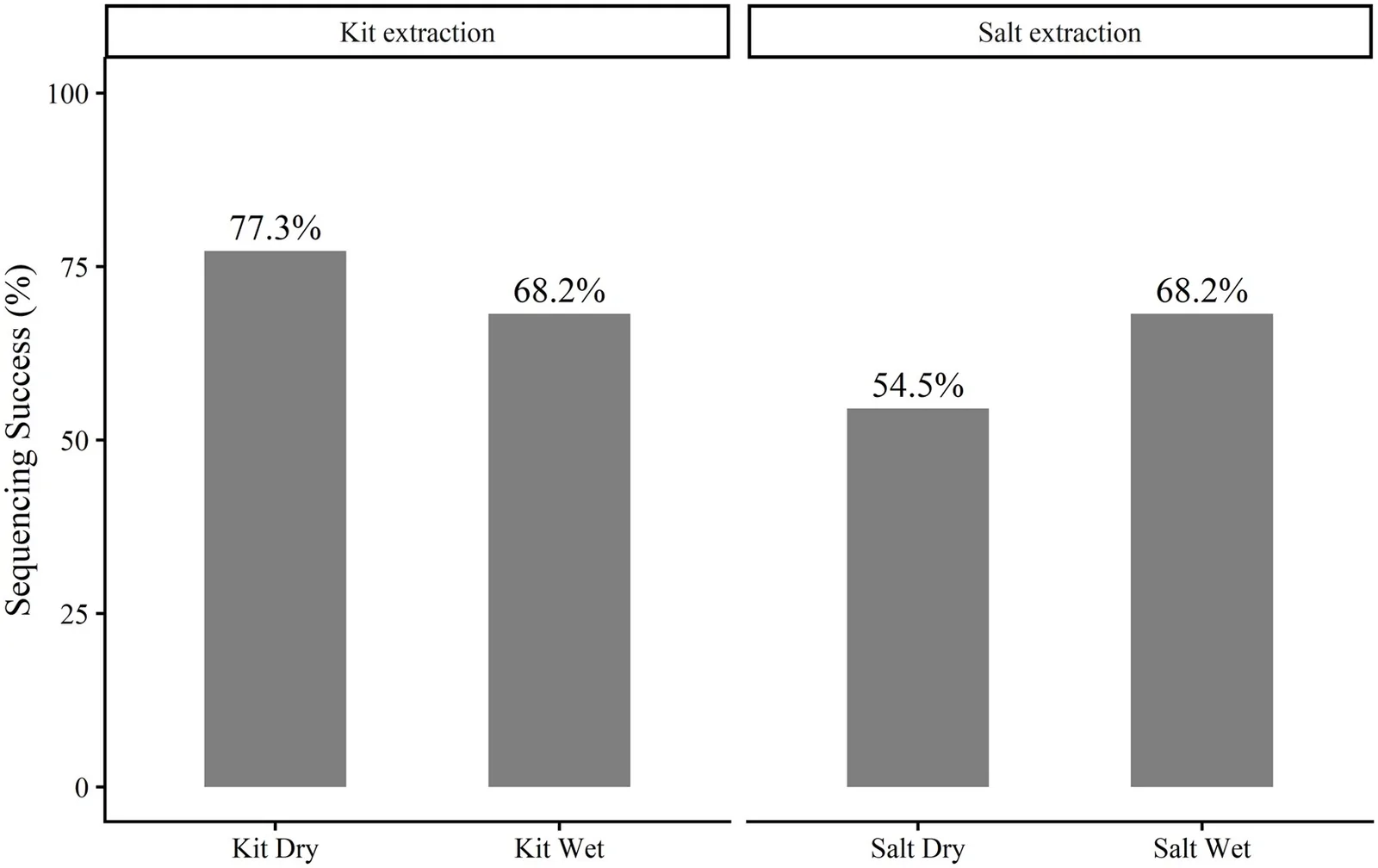
Sequencing success (%) of DNA samples of salt-extracted dry vs wet storage and kit-extracted dry vs wet storage.

Among the salt-extracted wet-storage samples, 20 out of 22 samples successfully amplified on the gel (90.91%), and 15 out of 22 gave clean sequencing reads (68.18%). Similarly, under salt-extracted dry storage, 19 out of 22 samples successfully amplified on the gel (86.36%), and 12 out of 22 samples yielded clean sequencing reads (54.54%). Among the kit-extracted wet storage samples, 17 out of 22 samples successfully amplified on the gel (77.27%), and 15 out of 22 samples yielded clean sequencing reads (68.18%). Similarly, under salt-extracted dry storage, 20 out of 22 samples successfully amplified on the gel (90.91%), and 17 out of 22 samples yielded clean sequencing reads (77.27%; Fig. 13).

Comparison among endemic anuran genera from the Western Ghats biodiversity hotspot showed that samples from the *genus Nyctibatrachus* yielded the maximum clean reads. Out of 28 samples, 20 samples gave clean reads (71.42%), whereas 14 samples out of 22 from the *Pedostibes* genus (63.63%) and 12 samples out of 20 from *Indirana* genus were successful (60%). In the urban areas, the genus Duttaphrynus had 8 samples successfully sequenced out of 10 (80%). From the genera *Euphlyctis* and *Minervarya*, only two individuals were sampled and sequenced, and both gave clean reads, whereas from the genus *Microhyla,* two out of the four samples were sequenced successfully.

### DNA concentration and snout-to-vent length

**Figure 14:**
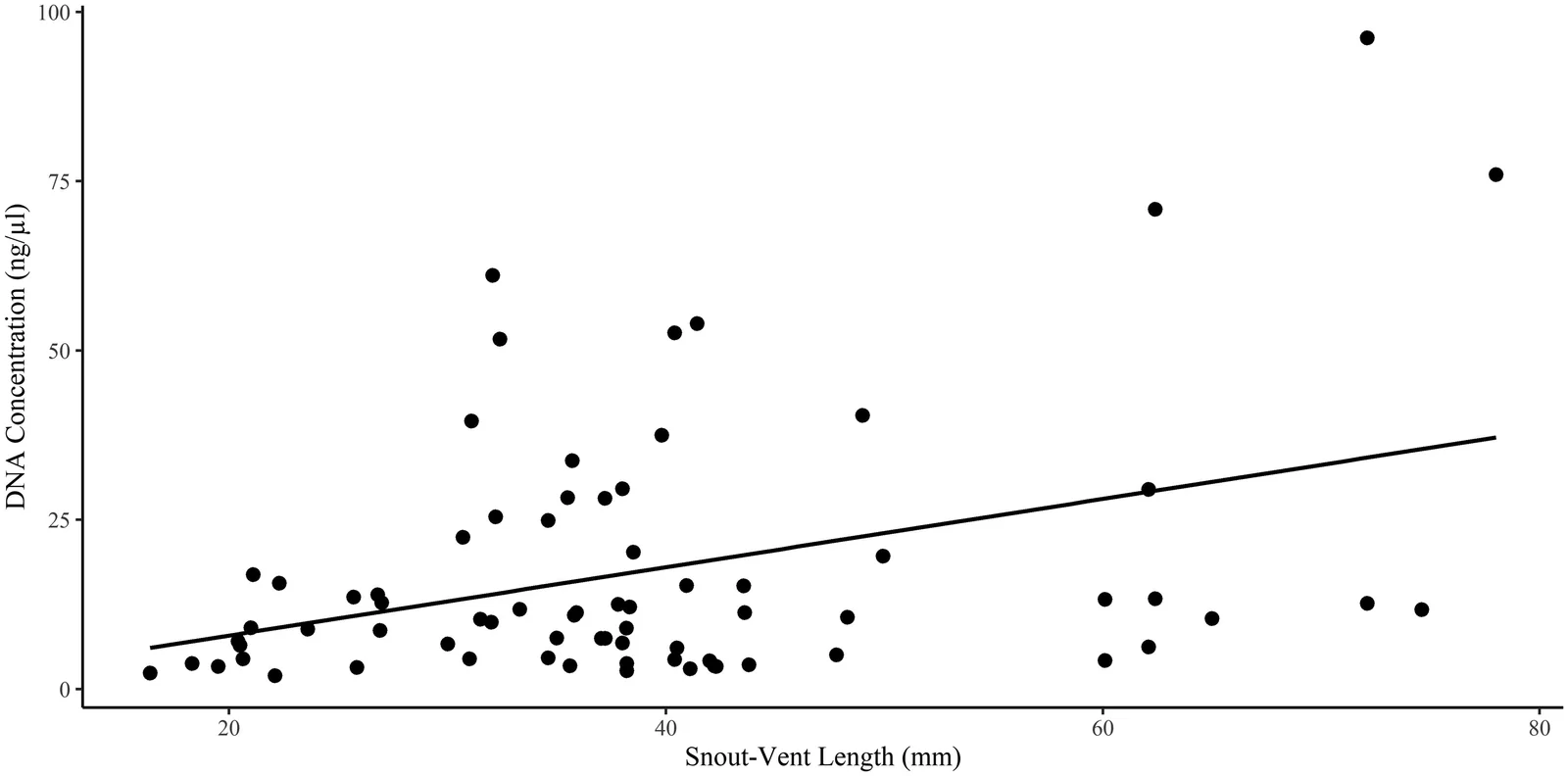
Scatterplot representing the relationship between SVL length (mm) and DNA concentration (ng/μl) obtained from skin swab samples.

This study included anurans with snout-to-vent length (SVL) ranging from 13.1 mm to 78 mm. We assessed the relationship between SVL (mm) and DNA concentration (ng/µl) and observed a weak positive relationship with a regression coefficient of *R*^2^ = 0.14; *P* < 0.05 (Fig. 14).

## Discussion

Our study demonstrates the feasibility of using minimally invasive skin swab samples to obtain DNA across a broad taxonomic range of anurans. Successful amplification across all seven genera indicates that this method can be useful in anuran genetic studies, offering a minimally invasive alternative to traditional tissue sampling, which can be lethal or stressful to individuals [15]. The consistent amplification across species highlights the utility of skin swabs for molecular studies in amphibians [18, 26].

### Limitations, challenges, and future directions

The fundamental challenge in amphibian skin sampling is that it may contain high levels of environmental contaminants, skin alkaloids, and microbial DNA [26] that hinder DNA recovery or downstream sequencing; however, we did not observe contamination from skin alkaloids or microbiota in our sequencing results. In our study, clean sequence reads showed 98%–99% sequence similarity in BLAST results, confirming species identity for further phylogenetic analyses. This study shows that skin swabbing can yield DNA of sufficient quality and can be utilised for taxonomy and genetic studies. Future studies should also test the efficacy of DNA recovered from skin swabs using next-generation sequencing methods, which typically require large amounts of DNA for downstream processes.

Although we did not compare skin swabs with cloacal or buccal swabs, the consistent results across multiple amphibian species show that the minimally invasive skin swabbing method can be used for genetic studies on protocol alterations on a case-by-case basis. A study by Pichlmüller *et al.* [22] suggested that the DNA quality and quantity obtained can vary depending on the type of swab used. The swab samples were collected by different individuals on the study team, which could have led to differences in swabbing procedures, although all of them used the same swab material and performed 60 strokes. Studies advocate handling-induced stress using skin, buccal, and cloacal swabs in amphibians [33, 34]. For this reason, toe-clipping is a preferred method as this involves less handling time. The key parameters, such as infection under tropical conditions following toe-clipping (or amputation) in the natural environment, and their impact on individual fitness and long-term effects on populations, are lacking. In our study, we reduced handling time and observed that, upon release, individuals began croaking, suggesting that skin swabbing may not have caused adverse effects. We emphasise that skin swabbing in anurans should be handled on a case-by-case basis, as results vary across anuran genera, as observed in our results. Thereby, we highlight the need for potential pilot studies with large sample sizes across species, genera, and families, and across various landscapes globally, to assess the efficacy of the skin swabbing method. Future studies should investigate the optimal stroke length and the minimum number of strokes to clarify swabbing efficacy, thereby standardising the method based on skin texture and moisture content. Tests are also needed to find the effect of swab material on DNA yield, as tested by Martin *et al*. [17], as the type of swab material can influence DNA quantity.

### DNA yield comparisons

So far, studies on amphibian skin swab sampling have yielded mixed, generally cautionary, results regarding DNA yield and quality. A study by Prunier *et al.* [15] showed that dorsal skin swabs yielded approximately 8.4 times less DNA than buccal swabs in alpine newts. Despite lower yields, the authors recommended skin swabbing as an alternative to buccal swabbing for vulnerable and small individuals, as it uses less handling time. Based on the literature, buccal swabbing can occasionally lead to buccal lesions with bleeding, whose short- and long-term consequences are unknown (infection risk, feeding, fitness) [14, 19]. Such adverse effects are not seen in the skin swabbing method, as per our experience and observations. Several studies have shown that skin swabbing yields less DNA and low genotyping success [17, 23]. However, our study shows that skin swabbing can be utilised for basic genetic studies on multiple amphibian species.

### Comparative evaluation of DNA extraction and swab preservation methods: salt vs. kit and dry vs. wet swabbing

In our study, the salt extraction method provided similar DNA concentrations as Qiagen™ kit extraction method. However, a limitation of our study was that DNA quantification was not carried out using a fluorometric method. A study by Martin *et al.* [17] showed that salt extraction and the Qiagen™ kit produced similar DNA yields, but the kit-extracted DNA showed higher purity, indicated by A260/230 and A260/280 ratios from fluorometric quantification of DNA, which was similar to our study results, although our study results were derived from the spectrophotometric quantification. Therefore, the DNA obtained from skin swabs in this study can be comparable with the DNA obtained from buccal swabs for downstream processes. Future research should also include fluorometric methods and compare them for accuracy and sensitivity.

This study is the first from the Indian tropical region to demonstrate the feasibility of DNA recovery by comparing extraction methods and storage conditions. Currently, there are no published studies from this region comparing storage conditions and DNA extraction techniques specifically for anuran skin swabs. Our results suggest that the extraction or storage method has no influence on DNA yield. In terms of DNA purity, the kit extraction method yielded higher purity than the salt extraction method. However, kit extraction methods can be expensive and may suffer DNA loss during column binding and washing steps, resulting in lower overall yields [35], whereas salt extraction methods are cost-effective and require minimal equipment, often yielding a sufficient amount of DNA from degraded or low-quality samples [17]. Our study also showed that dry swabs exhibit higher purity than wet swabs, irrespective of the extraction method. Overall, dry swabs can be a cost-effective and easy storage alternative, especially in remote field conditions. In the future, studies should test silica gel as a dry storage alternative, as used by Martin et al. [17] for long-term storage of non-extracted DNA.

### Body size and skin swabs

In amphibians, body size and skin surface area are positively correlated. Larger species have greater skin surface area, which may yield a larger quantity and higher-quality DNA. In a sample of 80 adults, we found a weak correlation between body size and DNA concentration (Fig. 14). Further research should include multiple samples to assess DNA recovery from skin swabs across body sizes. The efficacy of skin swabs is influenced not only by body size but also by skin type. For example, the study by Ringler *et al.* [23] indicated that skin swabs from dry-skinned dendrobatid frogs yield poor DNA quantity. On the contrary, we obtained good results with the Malabar tree toad, which has dry, warty skin (63% successful sequencing). This indicates that the efficacy of skin swabs can be species-specific and cannot be generalised.

Overall, this study implemented a functional methodology adapted to both heterogeneous climate and financial constraints typical of tropical countries. In conservation biology, a sampling method that is as minimally invasive as possible (skin swabbing), cost-effective (salting out), and adapted to tropical climates (dry storage) is particularly relevant to tropical amphibian biodiversity conservation. The results from this study can serve as a guide for minimally invasive DNA collection procedures that will benefit amphibian welfare and scientific studies in India.

## Supplementary Material

bpag040_Supplementary_Data
